# Enhanced Radiosensitization by Gold Nanoparticles with Acid‐Triggered Aggregation in Cancer Radiotherapy

**DOI:** 10.1002/advs.201801806

**Published:** 2019-01-08

**Authors:** Yumin Zhang, Fan Huang, Chunhua Ren, Jinjian Liu, Lijun Yang, Shizhu Chen, Jinglin Chang, Cuihong Yang, Weiwei Wang, Chuangnian Zhang, Qiang Liu, Xing‐Jie Liang, Jianfeng Liu

**Affiliations:** ^1^ Tianjin Key Laboratory of Radiation Medicine and Molecular Nuclear Medicine Institute of Radiation Medicine Chinese Academy of Medical Sciences & Peking Union Medical College Tianjin 300192 P. R. China; ^2^ CAS Center for Excellence in Nanoscience CAS Key Laboratory for Biomedical Effects of Nanomaterials and Nanosafety Chinese Academy of Sciences and National Center for Nanoscience and Technology of China Beijing 100190 China; ^3^ Tianjin Key Laboratory of Biomaterial Research Institute of Biomedical Engineering Chinese Academy of Medical Science and Peking Union Medical College Tianjin 300192 P. R. China; ^4^ University of Chinese Academy of Sciences Beijing 100049 P. R. China

**Keywords:** aggregation, gold nanoparticles, pH responsiveness, radiosensitizers, tumor retention

## Abstract

An ideal radiosensitizer holding an enhanced tumor retention can play an incredible role in enhancing tumor radiotherapy. Herein, a strategy of acid‐triggered aggregation of small‐sized gold nanoparticles (GNPs) system within tumor is proposed and the resulting GNPs aggregates are applied as a radiosensitizer in vitro and in vivo. The GNPs system with the acid‐triggered aggregation achieves an enhanced GNPs accumulation and retention in cancer cells and tumors in the form of the resulted GNPs aggregates. As a consequence, the radiosensitization effect shows significant improvement in cancer radiotherapy, which is shown in the studies of DNA breakage and the comet assay, and the sensitizer enhancement ratio (SER) value of the GNPs system (1.730) with MCF‐7 cancer cells is much larger than that of the single GNPs (1.16). In vivo antitumor studies reveal that the GNPs system also enhances the sensitivity of MCF‐7 tumor xenograft to radiotherapy. Furthermore, the GNPs aggregates improve the signal of small GNPs in vivo photoacoustic imaging. This study provides a new strategy and insights into fabricating nanoaggregates to magnify the radiosensitive efficiency of nanosystems in cancer radiotherapy.

Radiotherapy (RT)^1^ is an irreplaceable treatment strategy for effectively controlling local tumor and eradicating unresectable parts of tumor in current clinics, which has been mostly applied for combining with chemotherapy and surgical therapy. With respect to killing cancer cells, it is much unquestionable to need a high‐energy dose of ionizing radiation, but the severe radiation damage for adjacent healthy tissues cannot be ignored.^2^ More troubling, when reducing the radiation dose or increasing the radiation times, it would induce the emergence of radiation resistance of cancer cells, which could remarkably lower the radiotherapeutic efficiency and ultimately lead to the failure of RT.^3^ To overcome these limitations, a good deal of radiosensitizers which can raise the sensitization of cancer cells for RT have been developed and widely used in the clinical treatments, such as sodium glycididazole and sanazole.^4^ However, physical toxicity and severe side effects generated by these drugs cannot be adapted or tolerated by patients during the RT. Therefore, it is urgent to develop an ideal radiosensitizer which possess a number of properties including i) excellent biocompatibility, ii) enhanced tumor accumulation and retention, and iii) rapid renal clearance once distributed into other organs, thereby boosting the radiotherapeutic efficiency and specificity for tumors as well as minimizing the radiation damage for normal tissues.^5^, ^6^

In recent years, it has been shown that nanomaterials hold promising prospects in enhanced radiotherapeutic strategies because of their superiorities in biomedical applications.^6^, ^7^ Particularly, gold nanoparticles (GNPs),^8^ high‐Z materials with strong X‐ray or gamma‐ray attenuation capability, can be used as a radiosensitizer to precipitate the radiation energy within tumors and improve the radiotherapeutic efficiency. Meanwhile, good biocompatibility and facile size controlling of GNPs make it possible for an ideal radiosensitizer. However, there is a huge challenge for GNPs to enhance RT that it must meet the needs of the rapid clearance in vivo and long‐term tumor retention at the same time to improve the radiotherapeutic specificity and maximize the radiosensitive efficiency. It is well known that the different size of nanomaterials in the course of drug delivery showed various characteristics: ≈100 nm of nanomaterials displayed longer blood circulation and tumor accumulations, but failed in infiltrating into tumors, which was because their size is too large to permeate the dense structures of tumors. As for the small‐sized nanomaterials with ≈30 nm, they are good at tumor infiltration and have short‐lived blood circulation property which fulfilled the attributes of rapid clearance mentioned above for an ideal radiosensitizer.^9^, ^10^ However, the small‐sized nanomaterials that penetrated into tumors could still flow back into blood circulation or diffuse into the surrounding tissues, thus resulting in their depletion within tumors and increase within adjacent tissues.^11^, ^12^

On the basis of the excellent tumor penetration and rapid clearance of small‐sized nanomaterials, it is very promising that the ≈30 nm GNPs can be developed as potential radiosensitizers to solve the problem of backflow and random diffusion of the retained GNPs within tumors.^10^ Therefore, we hypothesize that if the ≈30 nm GNPs can assemble or aggregate into a larger‐sized aggregates after penetrating into tumors, and it will be trapped into the tumor site in the form of GNPs aggregates and prevent the backflow and random diffusion, thus greatly enhancing the retention of GNPs within the tumor. Exhilaratingly, tumor microenvironments (acidic milieu, high GSH concentration, and over‐expressed enzyme of cancer cells) provide an attractive opportunity to achieve this goal.^13^ Liang and co‐workers^14^ prepared a caspase 3‐instructed aggregation of Fe_3_O4@1 NPs by using an enzyme‐instructed condensation reaction, which hold a specificity for T_2_ enhanced MR imaging in tumor apoptosis and might be applied to detect the chemotherapeutic efficiency of tumors in routine preclinical studies in near future.

Nevertheless, there is still no report of using the acid‐triggered aggregation strategy for enhanced RT of cancer. Herein, we developed a GNPs system based on the acid‐triggered aggregation of ≈30 nm GNPs to serve as a novel radiosensitizer for enhanced RT (**Scheme** **1**). It consists of two kinds of GNPs that modified with Asp‐Asp‐Asp‐Asp‐Asp‐Cys peptide and Lys‐Gly‐Gly‐Lys‐Gly‐Gly‐Lys‐Cys peptide grafting 2,3‐Dimethylmaleic anhydride (DA), respectively, which was named as GNPs‐A and GNPs‐B. When arriving within tumors, the negative surface charge of GNPs‐B will be reversed into positively charged state in response to tumor pH, following the electrostatic interaction with negatively charged GNPs‐A and thus forming the GNPs aggregates with larger size.^15^ Therefore, the rapid blood clearance of small‐sized GNPs make this approach nontoxic to the body which is an important property for clinical application, and the formed GNPs aggregates can offer them a long‐term tumor retention. Most importantly, under the gamma radiation widely used in RT, the obtained GNPs aggregates in tumor can produce more reactive oxygen species (ROS) to damage the tumor cells, thereby enhancing the efficiency of RT.^16^, ^17^ In addition, the intensity of photo‐acoustic (PA) imaging for GNPs system will be also improved as its size increasement, benefiting the synchronous diagnosis during RT.^18^ Therefore, this GNPs system with acid‐triggered aggregation in tumor holds a great potential in the enhanced imaging‐guided tumor RT.

**Scheme 1:**
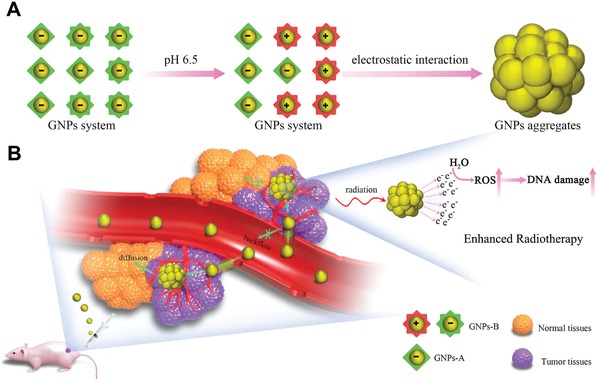
A) Diagram depicting the acid‐triggered aggregation and composition of GNPs system. The GNPs‐B undergoes a charge reverse when exposed to acidic environment, where electrostatic interaction further occurs with the GNPs‐A to form aggregates. B) Schematic illustrations of in vivo behavior of GNPs system after intravenous injection for increased tumor retention and enhanced RT.

The small‐sized GNPs were firstly synthesized by the citrate‐reduction method, and their diameters were about 28.2 nm, which was confirmed by dynamic light scattering (DLS) and transmission electron microscope (TEM) (Figure S1, Supporting information). Subsequently, the purity and identity of these two peptides (Asp‐Asp‐Asp‐Asp‐Asp‐Cys peptide, peptide A and Lys‐Gly‐Gly‐Lys‐Gly‐Gly‐Lys‐Cys peptide, peptide B) we synthesized were confirmed by liquid chromatograph‐mass spectrometer (LC‐MS) (Figures S2 and S3, Supporting information). Thirdly, DA was grafted onto the side chain of peptide B through the reaction between carboxyl groups of DA and amino groups of peptide B, and ^1^H NMR results (Figure S4A,B, Supporting information) showed that side chain amino of Lys was successfully modified with DA by the chemical shift change from peak a to peak b.^15^ Upon incubation of DA‐grafted peptide B in the acidic medium (pH 6.5), the peak b could shift back to peak a in the ^1^H NMR result (Figure S4C, Supporting information), indicating the occurrence of charge reverse of this compound through the sensitive hydrolysis of dimethylmaleic amide under the mildly acidic environment. Then, GNPs‐A and GNPs‐B was prepared through metal coordination of “Au‐S” between GNPs and peptide A or DA‐grafted peptide B, respectively. Finally, the GNPs system was achieved by the mixture of GNPs‐A and GNPs‐B (1:1). As shown in **Figure** **1**A, the absorption peak of GNPs system generated a feeble red shift in ultraviolet visible absorption spectrum after being modified with above two peptides, which can further prove the successful construction of GNPs system. The average size of GNPs system was measured to be about 32 nm with a spherical structure (Figure 1B,C). Moreover, the time‐dependent measurement of DLS and UV–vis spectra of this GNPs system confirmed its preferable stability in phosphate buffer solution (PBS) (pH 7.4) after incubation for 24 h (Figure S5A,B, Supporting information). After preparing the GNPs system, we then investigated its property of acid‐triggered aggregation in vitro. As shown in Figure 1E, the size of GNPs system immediately increased from 32.6 to 912.8 nm after incubation for 5 min at mildly acidic environment (pH 6.5), and even increased to 1468 nm after incubation for 30 min. It could be clearly observed that large GNPs aggregates were formed when the GNPs system encountered acidic environment (Figure 1F). Meanwhile, as shown in Figure 1D, GNPs system displayed obvious red shift of ultraviolet absorption peak at pH 6.5 and the spectrum became wider, further verifying the implementation of the strategy of acid‐triggered aggregation. Meanwhile, as the acidic condition triggered the detachment of DA, and the GNPs aggregates formed subsequently. Because the DA could not re‐conjugate into the side chain of peptide B in pH 7.4, the aggregates is stable in neutral pH. As shown in Figure S6 in the Supporting information, the UV–vis spectra of GNPs aggregates showed that it hold a preferable stability in PBS solution (pH 7.4). And the distinct variation of zeta potential of GNPs‐B also demonstrated that it had acid‐triggered charge–reversal property and could electrostatically interacted with GNPs‐A, which realized acid‐triggered aggregation of them (Figure S7, Supporting information). In contrast, the size and UV‐vis spectra of GNPs‐A or GNPs‐B had no differences with the change of pH values (Figures S8 and S9, Supporting information), which could be further explained that the aggregating specificity of GNPs system.

**Figure 1:**
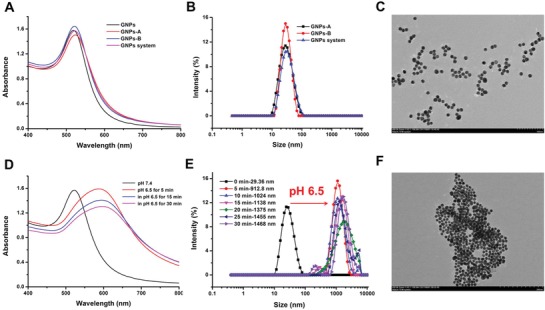
The characterization of GNPs system at pH 7.4 including A) UV–vis spectra, B) size distribution, and C) TEM image, and the aggregating behavior of GNPs system at pH 6.5 including D) UV–vis spectra, E) size distribution, and F) TEM image.

Having confirmed the ability of GNPs system for acid‐triggered aggregation in solution, we next investigated whether this property could be achieved in cellular levels. The cellular uptake of GNPs system was studied in MCF‐7 cells by using confocal microscope which was based on the green fluorescence of FITC‐PEG_1000_‐SH labeled GNPs system. As shown in Figure S10A in the Supporting information, the fluorescence intensities of individual GNPs‐A or GNPs‐B were very low, showing less GNPs was internalized by MCF‐7 cells. However, the intracellular green fluorescence intensity of GNPs system was significantly increased and made much stronger than that of individual GNPs‐A or GNPs‐B groups, which suggested that the GNPs system aggregated together, triggered by acidic environment of cancer cells, and resulted in stronger fluorescence intensity. Meanwhile, the results of flow cytometry (Figures S10B and S11, Supporting information) also showed the similar tendency with above observation. Moreover, this enhanced cellular uptake behavior of GNPs system showed time‐dependent and dose‐dependent manner (Figure S10C,D, Supporting information). In addition, the cellular uptake was also studied by TEM (Figure S10E–G, Supporting information). It could be clearly seen that a lot of GNP aggregations were located in the cytoplasm for GNPs system group, and only indistinct particles were observed for individual GNPs‐A or GNPs‐B groups, which further confirmed that the GNPs system possessed acid‐triggered aggregation property and could enhance the cellular uptake of GNPs.

After achieving the acid‐triggered aggregation of GNPs system both in solution and in cellular level, we investigated the radiosensitization of GNPs system in vitro. Firstly, the pictures which were shown in **Figure** **2**A intuitively indicated the degree of colony formation of various GNPs groups were obviously inhibited as compared to RT only (the result of control group treated without RT was shown in Figure S12, Supporting information). Obviously, GNPs system group displayed the best inhibition ability for colony formation, compared with RT only, GNPs‐A and GNPs‐B groups. Meanwhile, as shown in Figure 2B, the survival fraction curves of GNPs system under different dose radiation displayed a much stronger radiation enhancement effect compared with that of control groups treated with RT. Moreover, the inhibition efficiency of colony formation for GNPs system under 4 Gy was equivalent to that of RT only under 6 Gy, indicating that usage of GNPs system could decrease the radiation dose. In addition, the SER10 value of GNPs system calculated by the survival fraction curves was 1.730, which was much higher than that of the single GNPs‐A or GNPs‐B (1.16 and 1.17). The SER10 value of GNPs system (1.730) was much higher than that of aggregation‐induced emission luminogen (1.62), and higher than that of GNPs (1.62), gold nanospikes (1.37), and gold nanorods (1.21) in the published works.^19^, ^20^ These data suggested that the SER10 value of GNPs was significantly improved because of the acid‐triggered aggregation of GNPs system.^17^, ^19^ Moreover, this enhanced inhibition efficiency for colony formation of GNPs system also showed concentration‐dependent manner (Figure S13, Supporting information). The SER10 value of GNPs system at 50 and 100 µg mL^−1^ calculated by Figure 2C was 2.09 and 2.13, respectively, which further boost the sensitive efficiency as compared with of 20 µg mL^−1^. The inconspicuous difference of GNPs system for SER value between 50 and 100 µg mL^−1^ can be explained by that the size of GNPs aggregation could not be infinitely increased even in the acidic medium, which has been confirmed in Figure 1.

**Figure 2:**
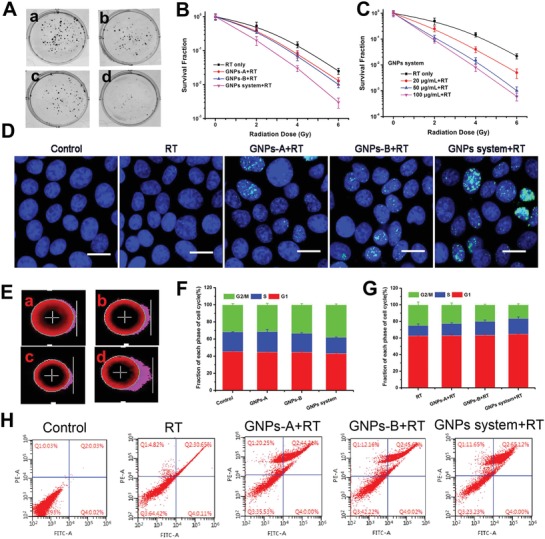
In vitro sensitization efficiency study of GNPs system. Colony formation curves of MCF‐7 cancer cells received various GNPs treatments indicated treated with 6 Gy (A, a: RT only, b: GNPs‐A+RT, c: GNPs‐B+RT, and d: GNPs system+RT). B) Colony formation curves of MCF‐7 cancer cells received various GNPs formulations treated with 0, 2, 4, and 6 Gy. C) Colony formation curves of MCF‐7 cancer cells received various GNPs system concentration treatments treated with 0, 2, 4, and 6 Gy. D) Immunofluorescent imaging of γ‐H2AX foci in MCF‐7 cells incubated with various GNPs treatments under 4 Gy irradiation, scale bar = 25 µm. Imaging of DNA fragmentation by using comet assay after CASP analysis in MCF‐7 cells with different GNPs treatments under 4 Gy irradiation (E, a: RT only, b: GNPs‐A+RT, c: GNPs‐B+RT, and d: GNPs system+RT). Cell cycle distribution histograms of MCF‐7 cells treated with various GNPs for 24 h F) without or G) with 4 Gy irradiation. The data were obtained by flow cytometry using cell cycle detection kit. H) Apoptosis ratios of MCF‐7 cells treated without (control) and with RT only, GNPs‐A+RT, GNPs‐B+RT, and GNPs system+RT after 4 Gy irradiation determined by flow cytometry.

To study the enhanced radio‐sensitization of GNPs system from radio‐sensitizing mechanism, the double‐strand breaks (DSBs) of DNA inside cancer cells were monitored by γ‐H2AX staining to appraise the efficiency of DNA damage during RT.^21^ As shown in Figure 2D, only weak green fluorescent spots were observed inside cell nucleus under 4 Gy RT only. However, in the presence of GNPs‐A or GNPs‐B under 4 Gy radiation, more green fluorescence was found in the cell nucleus which meant that the DSBs of DNA induced by RT was clearly exacerbated by the existence of GNPs. Most importantly, there were much higher levels of DSBs of DNA within the nuclei of cells in the case of GNPs system, and cell nucleus were overlaid with the green fluorescence in some MCF‐7 cells, indicating severe DNA damage in those cells. Afterward, the comet assay was employed to detect DNA damage in individual cell, where the tail length of cells showed the degree of DNA damage. As shown in Figure 2E, compared with control group (Figure S14, Supporting information), the cells in the radiation groups displayed different levels of tail length, and the GNPs system group showed longest tail length of cells in the four radiation groups, which suggested the most serious damage of DNA. Hence, this enhanced sensitivity effect of GNPs system coincided well with colony formations study above.

In addition, the cell cycle distribution study was applied to further evaluate the potential mechanism of GNPs‐induced radio‐sensitizing effects. After being incubated with GNPs system or GNPs‐A (GNPs‐B), the cell cycle presented a subtle change which compared to control group (Figure 2F). Briefly, these three GNPs groups induced mildly more cells to distribute in the G2/M phase and less cells distribution in G_1_ phase and S phase than that of the control group. Owing to the most sensitive to radiation of the G2/M phased cells, the cells incubated with GNPs might be easily killed by RT, and the cell‐killing efficiency of GNPs system might be highest because that the most cells distributed in the G2/M phase.^22^ After 4 Gy radiation, the cells in the G2/M phase of all RT groups obvious reduced and the amount of the cells in the G1 phases relatively increased, and GNPs system showed the least cells in G2/M phase compared with other three RT groups (Figure 2G and Table S1, Supporting information). Therefore, GNPs systems showed the strongest cell‐killing efficiency and might be acted as a promising radiosensitizer during RT.^20^ Lastly, in order to estimate the GNPs system‐induced cell apoptosis ratios after 4 Gy irradiation, flow cytometry was carried out using Annexin V‐FITC/PI apoptosis detection kit. Comparing with the control group, all the irradiated groups showed different level of apoptosis. The cells apoptosis ratio of single GNPs groups were higher than that of RT only group, and the cells treated with GNPs system exhibited the highest total apoptosis ratio of 65.12% (Figure 2H).

After achieving the desirable radiosensitive effect of GNPs system by using the acid‐triggered aggregation in vitro, we then evaluated its functions in vivo, including pharmacokinetics, tissue distribution, toxicity, and tumor retention. All animal experiments were performed according to the protocol approved by Chinese Academy of Medical Science and Peking Union Medical College and following the Guiding Principles under the Care and Use of Animals of the American Physiological Society. Firstly, the GNPs system showed a rapid blood clearance and a similar curve to that of GNPs‐PEG2000 (**Figure** **3**A), which might be attributed to their similar particle size and zeta potential under pH 7.4 condition (Figure S15A,B, Supporting information). In contrast, the tissue distribution of these two GNPs in reticuloendothelial system (RES) organs showed a significant difference after 24 h injection (Figure 3B). The accumulation of GNPs system in liver and spleen was obviously lower than that of GNPs‐PEG2000. This similar blood clearance but different accumulation in RES system between GNPs system and GNPs‐PEG2000 meant that the GNPs system have more chance to enter into tumor site, and probably because that the negative charge of peptide on the GNPs system refused to the phagocytosis of RES organs. Besides, the Au concentration in kidney were higher than that of heart and lung for both GNPs system and GNPs‐PEG2000, which further proved that they suffered from a rapid renal clearance and led to a lower Au concentration in the lung and heart.^23^, ^24^ Additionally, for the groups treated with GNPs system and GNPs‐PEG2000, the HE staining results showed no obvious histopathological abnormalities and lesions in liver, spleen, and kidney (Figure 3C). Meanwhile, the main results of hematology and blood biochemistry depicted in Figures S16 and S17 in the Supporting information also showed an indistinctly physical toxicity. These observations can be explained as follows: the higher biocompatibility of GNPs system itself (Figure S18, Supporting information), the faster blood clearance in vivo, and the lower accumulation in RES organs.

**Figure 3:**
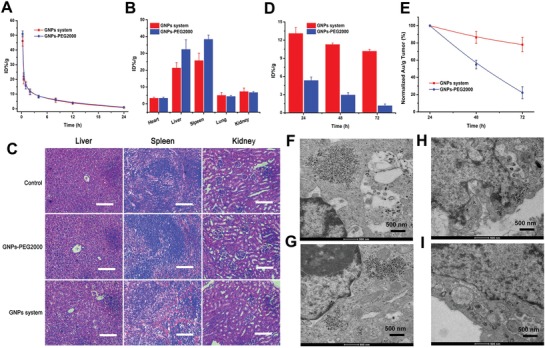
In vivo evaluation of GNPs system. A) Blood circulation curves and B) tissue distribution at 24 h injection of GNPs system and GNPs‐PEG2000 after intravenously injecting BALB/c mice. C) Representative HE stained images of major organs including liver, spleen, and kidney collected from the control untreated mice and GNPs‐injected BALB/c mice at 24 h post injection, scale bar = 50 µm. D) Accumulation of GNPs system and GNPs‐PEG2000 in MCF‐7 tumor in BALB/c nude mice at 24, 48, and 72 h post injection. E) Tumor uptake normalized at 48 and 72 h post injection to that at 24 h. Representative TEM images of sections of MCF‐7 tumor tissue after injection with GNPs system for F) 24 h and G) 72 h, and TEM images of sections of MCF‐7 tumor tissue after injection with GNPs‐PEG2000 for H) 24 h and I) 72 h.

In this study, as a novel sensitizer, an ideal accumulation and retention in tumors is of critical importance for GNPs system, which can play a vital role in improving radiotherapeutic efficiency based on the level of tumor. Hence, tumor retention of GNPs system was studied in BALB/c nude mice bearing MCF‐7 tumors at 24, 48, and 72 h post injection, respectively. As shown in Figure 3D, it was found that the tumor accumulation of GNPs system was more than twice as high as GNPs‐PEG2000 at 24 h post injection, and as time goes on, this difference was getting wider at 48 and 72 h post injection. Moreover, GNPs system group showed a large tumor clearance curve and even 80% of GNPs amounts were retained in tumors after 72 h post injection (Figure 3E). TEM analysis (Figure 3F and Figure S19, Supporting information) after 24 h injection further explained the observation that a larger‐sized GNPs aggregates was formed within acidic environment of tumors, and then prevented the outflow of GNPs from tumor tissues. After 72 h injection, GNPs aggregates formed by GNPs system were still retained in tumors (Figure 3G), suggesting a longer retention within tumors and a slow tumor clearance. However, the accumulation of GNPs‐PEG2000 at 72 h post injection reduced to only about 20% compared with 24 h post injection (Figure 3D), and only few GNPs could be observed in the tumors under the TEM after 24 h injection (Figure 3H) but hardly could be seen at 72 h post injection (Figure 3I).^11^, ^18^ The rapid tumor clearance curve (Figure 3E) further proved that the small GNPs retained in tumors after 24 h might migrate into surrounding tissues or re‐enter the bloodstream from the tumor, and then depleted the GNPs reserved within tumors.^12^

Owing to the high density and extinction coefficients, GNPs can be used as contrast agents for PA imaging.^25^ Interestingly, we found that the GNPs aggregates formed by GNPs system at acidic environment could lead to an increasing absorption in near‐infrared region between 650 and 900 nm (Figure S20, Supporting information). Thus, we were encouraged to examine whether the PA imaging effect could be improved by the GNPs aggregates. Firstly, the in vitro PA imaging of GNPs system (90, 45, 22.5 µg mL^−1^) at pH 7.4 and pH 6.5 was studied by PA imaging system, and GNPs‐PEG2000 as the control. As shown in **Figure** **4**A and Figure S21 in the Supporting information, both GNPs system and GNPs‐PEG2000 showed enhanced PA signals with increased concentration, and the PA signals of GNPs‐PEG2000 at pH 6.5 showed similar intensity to that at pH 7.4, which was due to the similar UV–vis absorption in near‐infrared region between pH 7.4 and pH 6.5 (Figure S20, Supporting information). However, owing to the enhanced absorption of GNPs system at pH 6.5 as studied above, their PA imaging signals improved remarkably compared with that of pH 7.4, which was shown in Figure 4A,B. These results demonstrated that GNPs system could significantly enhance the imaging ability of GNPs under the specifically acidic environment of tumor and thus act as a good theranostic agent for tumor.^18^

**Figure 4:**
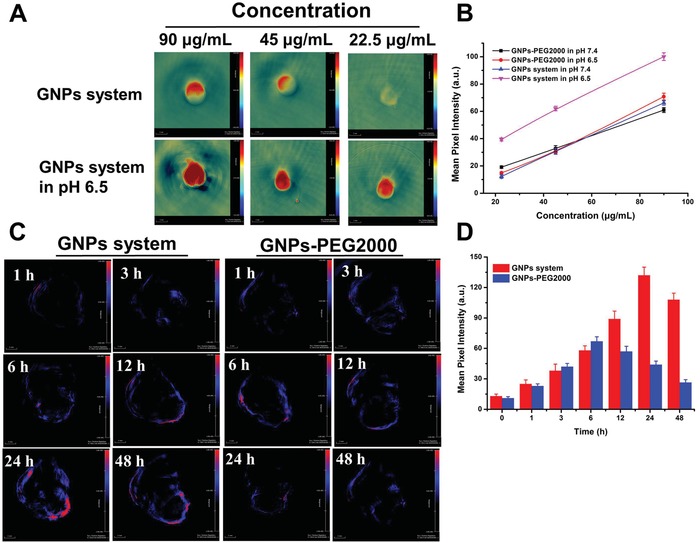
PA of GNPs system. A) The in vitro PA imaging of GNPs system at concentration of 90, 45, and 22.5 µg mL^−1^ at pH 7.4 and pH 6.5, respectively. B) The mean pixel intensity of PA signal measured from (A). C) The in vivo PA imaging of GNPs system and GNPs‐PEG2000 after 1, 3, 6, 12, 24, and 48 h intravenous injection, and D) PA intensity of tumor tissue treated with GNPs system and GNPs‐PEG2000 as a function of time.

Subsequently, their in vivo imaging effects were studied and the results were shown in Figure 4C,D. GNPs‐PEG2000 group showed a gradual tumor accumulation after injection and their PA signal reached the maximum intensity within tumor after 6 h (0 h of PA imaging was shown in Figure S22, Supporting information). Then their PA signal intensity gradually decreased, which meant that GNPs‐PEG2000 suffered from a tumor clearance after injection of 6 h. However, the GNPs system exhibited improved accumulated kinetics within tumors and they could stay at tumor tissue until to 24 h. Moreover, based on the longer tumor retention of the GNPs aggregates, the PA signals of GNPs system decreased slowly and obviously stronger than that of GNPs‐PEG2000 after injection of 12, 24, and 48 h, respectively. More importantly, the maximum PA signal of GNPs system appeared at 24 h after being injected, which contributed to optimize the radiation time and maximize their radiosensitive efficiency during RT.

Finally, the radiosensitive effects of GNPs system were studied in the MCF‐7‐luciferase tumor bearing mice model. As shown in **Figure** **5**A, all the radiation‐treated groups showed inhibition effect of tumor growth with different degrees compared with PBS group. Specifically, the GNPs‐PEG2000 group treated with 6 Gy radiation showed preferable antitumor efficiency compared with the 6 Gy RT only, indicating that the GNPs hold a radiosensitive property during the RT. While the GNPs system treated 6 Gy radiation group displayed the best antitumor efficiency in all of the radiation‐treated groups, suggested that GNPs system could significantly amplified radiosensitive efficiency due to the formation of GNPs aggregates triggered within the tumor, which allowed much more GNPs retained and accumulated together in the tumors than that of single GNPs‐PEG2000. Moreover, GNPs system group treated with 4 Gy radiation showed similar tumor inhibition efficiency with that of GNPs‐PEG2000 treated with 6 Gy radiation. This result revealed that using GNPs system could effectively reduce the radiation dose. In addition, the bioluminescent intensity of tumors by the bioluminescent imaging can reflect the proliferating activity of cancer cells.^26^ Before the treatment, as shown in Figure S23 in the Supporting information, tumors of mice of various groups showed a similar proliferating activity of the cancer cells. However, as shown in Figure 5C, tumors of mice of various groups showed a different level of proliferating activity after RT, in agreement with above data, GNPs system treated with 6 Gy had the lowest bioluminescent intensity and the best antitumor efficiency (Figure 5A,D).

**Figure 5:**
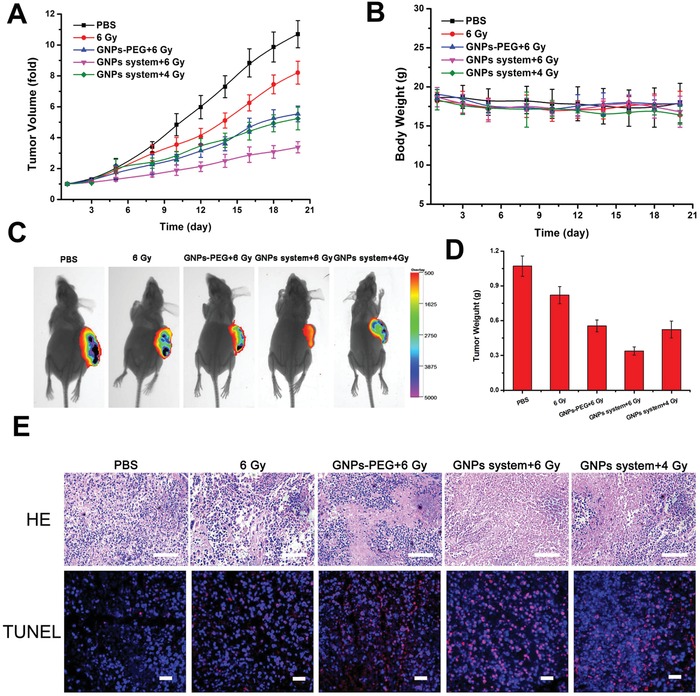
In vivo RT of GNPs system. A) Tumor volume growth curves and B) body weight curves of the mice after treatment with PBS, 6 Gy irradiation only, 6 Gy radiation after intravenous injection of GNPs‐PEG2000 for 24 h, and 6 or 4 Gy radiation after intravenous injection of GNPs system for 24 h. C) Bioluminescent imaging on MCF‐7‐luciferase tumor‐bearing mice 20 days after given indicated treatments. D) Tumor weight in the mice treated with various of treatment formulations at 20 days treatment. E) HE stained tumors (scale bar = 50 µm) and TUNEL assay (scale bar = 25 µm) in the mice treated with various treatment formulations at 20 days treatment.

Meanwhile, both of the tumor weights (Figure 5D), HE staining results, and TUNEL assays (Figure 5E) demonstrated that GNPs system could decrease tumor size and induce serious apoptosis and necrosis of the cancer cells during RT, further proving their desirable radiosensitive effect.^23^, ^27^ The weight changes of mice with different treatments were monitored during the course of RT. There was no obvious body weight change in all groups (Figure 5B), which indicated that GNPs system did not cause any side effects under 4 and 6 Gy radiation dose. Therefore, our strategy developed an excellent radiosensitizer with desirable radiotherapeutic effect and low side effect.

In summary, a novel tumor microenvironment sensitive radiosensitizer was developed based on the acidic‐induced GNPs aggregation system, which was composed of small GNPs modifying different charged peptide on the surface. Once arriving into the tumor with acidic pH, the surface charge of small GNPs with charge reversal property could be changed from negative to positive, resulting in the electrostatic interaction with another negatively charged GNPs and thus forming large‐sized GNPs aggregates. This GNPs aggregates have much longer tumor retention ability by blocking the migration and backflow of GNPs. Compared with small GNPs, the GNPs aggregates could significantly amplify the radiosensitive efficiency and improve SER10 value to reduce the radiation dose so as to alleviate the radiation damage for healthy tissues. At normal pH during circulation and distribution, the GNPs system showed a rapid blood clearance and a lower phagocytosis by RES system, leading to a radiosensitive specificity for tumors. Additionally, the GNPs aggregation could also improve the PA signal which was in favor of imaging‐guided enhanced RT for tumors. Therefore, the tumor microenvironment–triggered GNPs aggregation system we constructed is a promising strategy for the enhanced tumor RT.

## Conflict of Interest

The authors declare no conflict of interest.

## Supporting information

Supplementary
