# Supplementary material for: Enhanced Radiosensitization by Gold Nanoparticles with Acid‐Triggered Aggregation in Cancer Radiotherapy
Source: Adv Sci (Weinh). 2019 Jan 8;6(8):1801806. doi: 10.1002/advs.201801806 (PMC6469241; doi:10.1002/advs.201801806)
Supplement: Supplementary file 1 — Supplementary [file ADVS-6-1801806-s001.pdf]

Copyright WILEY-VCH Verlag GmbH & Co. KGaA, 69469 Weinheim, Germany, 2019.

ADVANCED SCIENCE
Open Access

Supporting Information

for *Adv. Sci.,* DOI: 10.1002/advs.201801806

Enhanced Radiosensitization by Gold Nanoparticles with Acid-Triggered Aggregation in Cancer Radiotherapy

Yumin Zhang, Fan Huang, Chunhua Ren, Jinjian Liu, Lijun Yang, Shizhu Chen, Jinglin Chang, Cuihong Yang, Weiwei Wang, Chuangnian Zhang, Qiang Liu, Xing-Jie Liang, and Jianfeng Liu**

Copyright WILEY-VCH Verlag GmbH & Co. KGaA, 69469 Weinheim, Germany, 2016.

Supporting Information

Enhanced Radiosensitization by Gold Nanoparticles with Acid-Triggered Aggregation in Cancer Radiotherapy

Yumin Zhang,[1] Fan Huang,[1] Chunhua Ren,[1] Jinjian Liu,[1] Lijun Yang,[1] Shizhu Chen,[2] Jinglin Chang,[1] Cuihong Yang,[1] Weiwei Wang,[3] Chuangnian Zhang,[3] Qiang Liu,[1] Xing-Jie Liang,[2, 4*] Jianfeng Liu.[1*]

Dr. Y. Zhang, F. Huang, C. Ren, J. Liu, L. Yang, J. Chang, C.Yang, Prof. Q. Liu, Prof. J. Liu

[1] Tianjin Key Laboratory of Radiation Medicine and Molecular Nuclear Medicine, Institute of Radiation Medicine, Chinese Academy of Medical Sciences & Peking Union Medical College. Tianjin 300192, P.R. China.

E-mail: lewis78@163.com

Dr. S. Chen, Prof. X.-J. Liang

[2] CAS Center for Excellence in Nanoscience, CAS Key Laboratory for Biomedical Effects of Nanomaterials and Nanosafety, Chinese Academy of Sciences and National Center for Nanoscience and Technology of China, Beijing, 100190, China.

E-mail: liangxj@nanoctr.cn

Dr. W. Wang, C. Zhang

[3] Tianjin Key Laboratory of Biomaterial Research, Institute of Biomedical Engineering, Chinese Academy of Medical Science and Peking Union Medical College, Tianjin 300192, P.R. China.

Prof. X.-J. Liang

[4] University of Chinese Academy of Sciences, Beijing 100049, P. R. China.

1. Supporting Figures

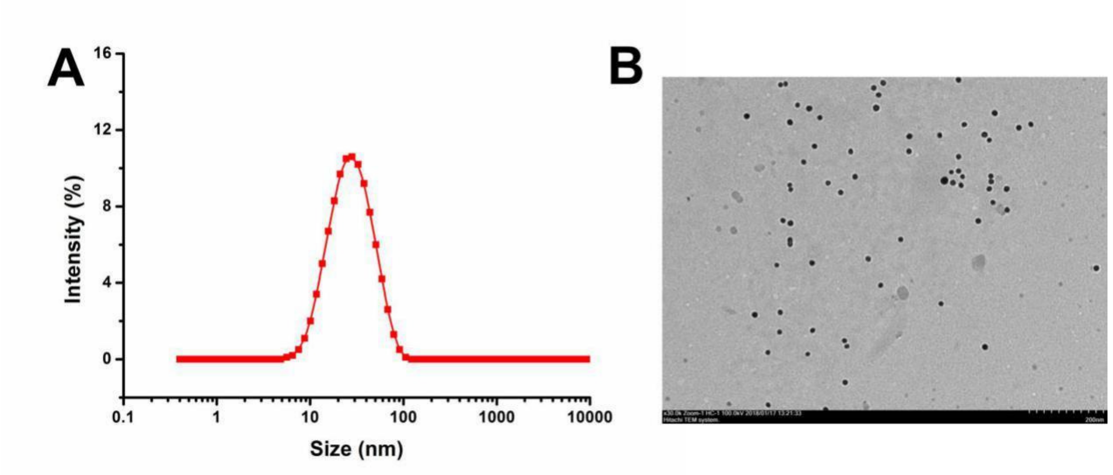

Figure S1. The size distribution and morphology of naked GNPs.

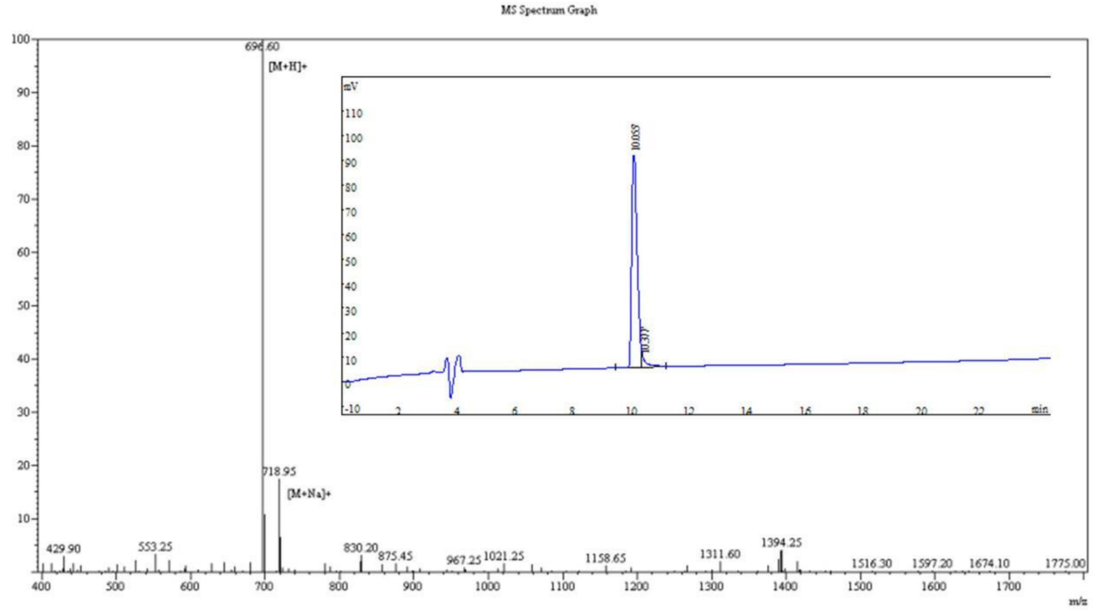

Figure S2. LC-MS spectrum of Asp-Asp-Asp-Asp-Asp-Cys peptide (peptide A).

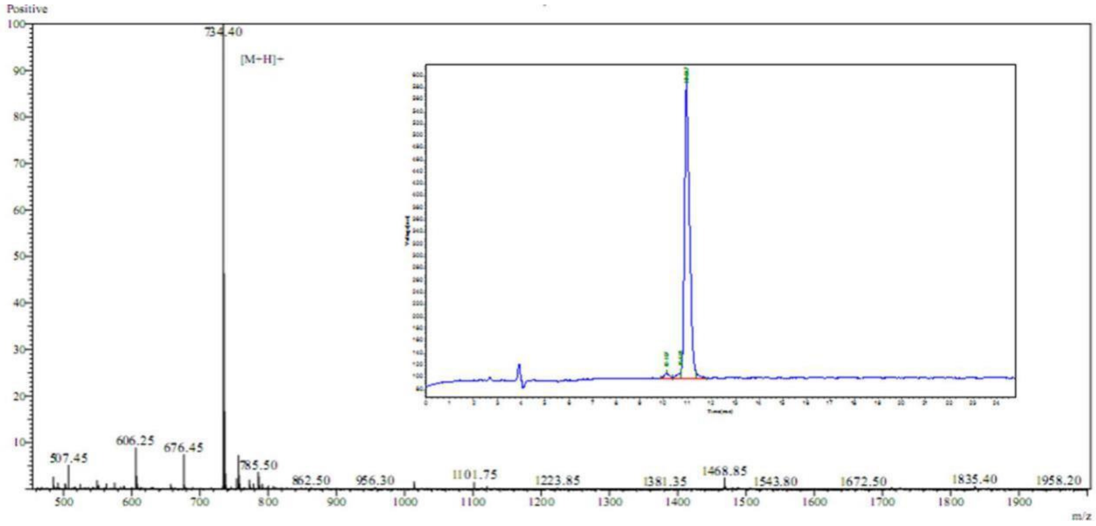

Figure S3. LC-MS spectrum of Lys-Gly-Gly-Lys-Gly-Gly-Lys-Cys peptide (peptide B).

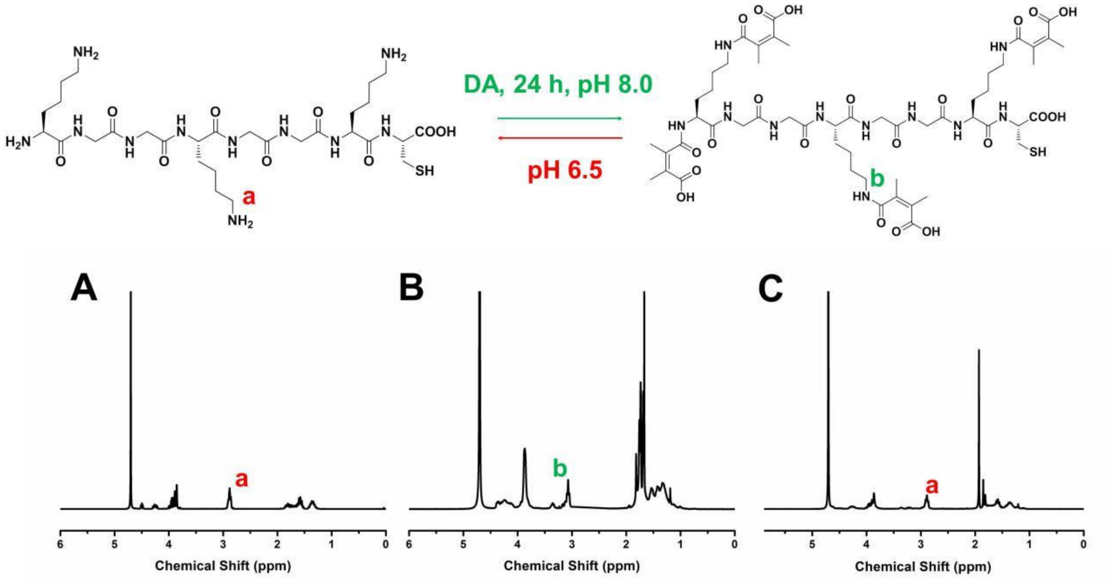

Figure S4. ^{1}H NMR results of peptide B (A) and DA grafted peptide B (B), and the ^{1}H NMR results of DA grafted peptide B after incubated at pH 6.5 (C) in D_2O/DCl.

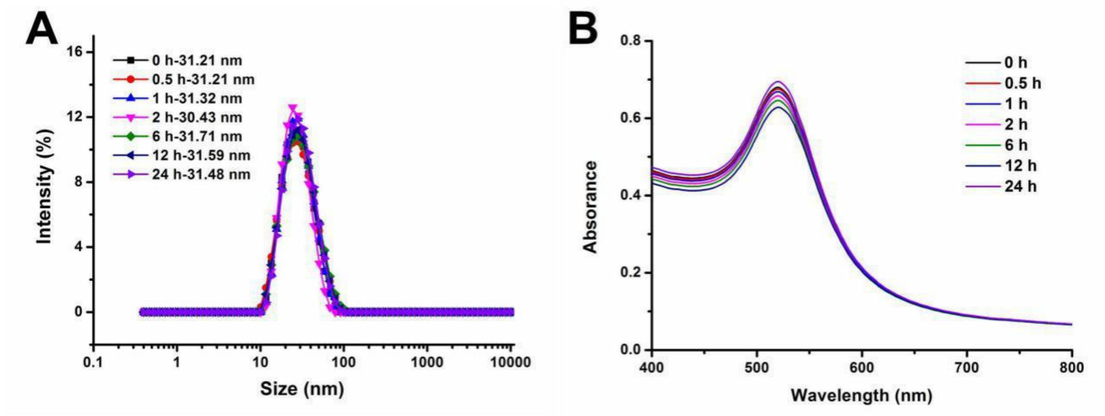

Figure S5. The time-dependent measurement of DLS and UV-vis spectra of GNPs system.

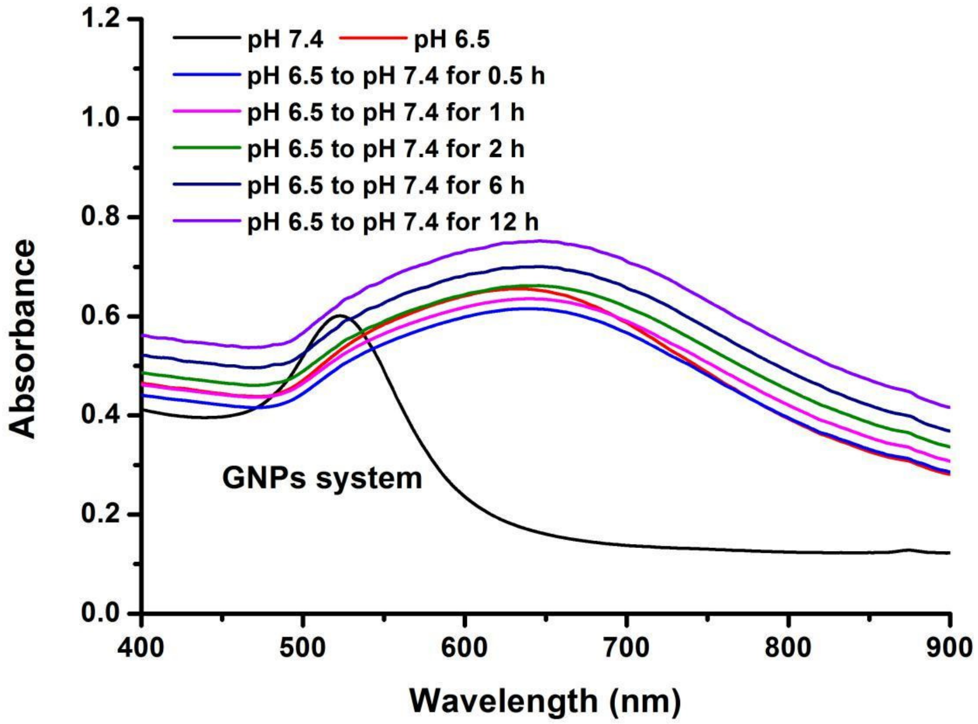

Figure S6. The time-dependent measurement of UV-vis spectra of GNPs system from pH 6.5 to pH 7.4.

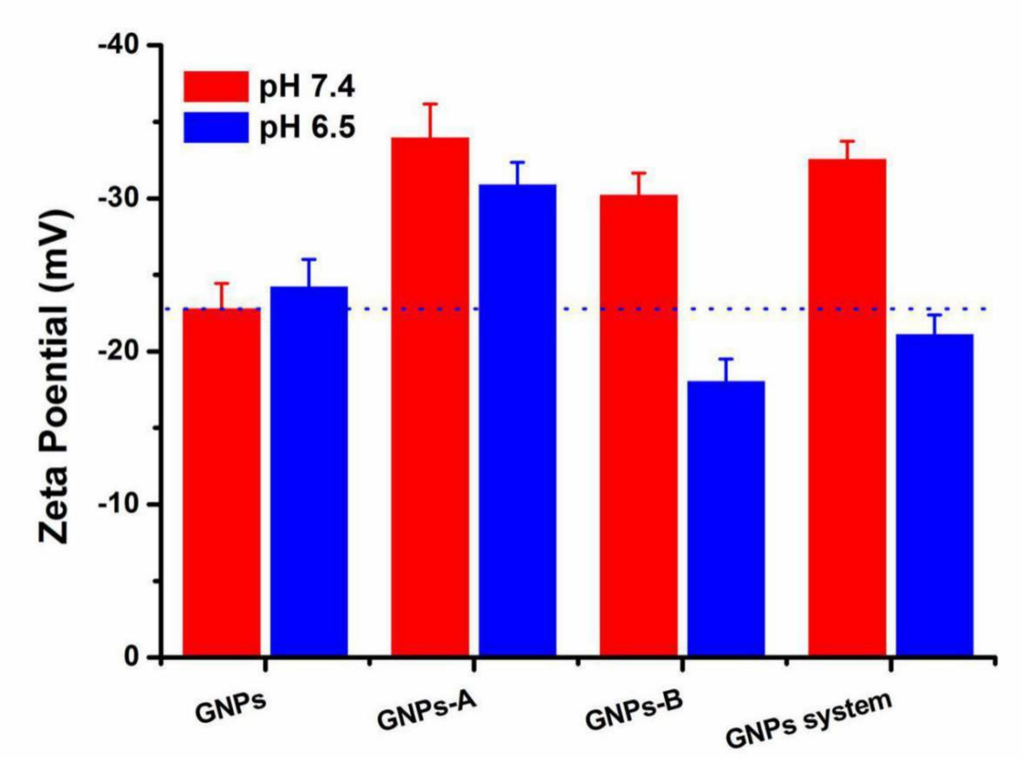

Figure S7. The zeta potential of various GNPs under 7.4 and 6.5 pH value.

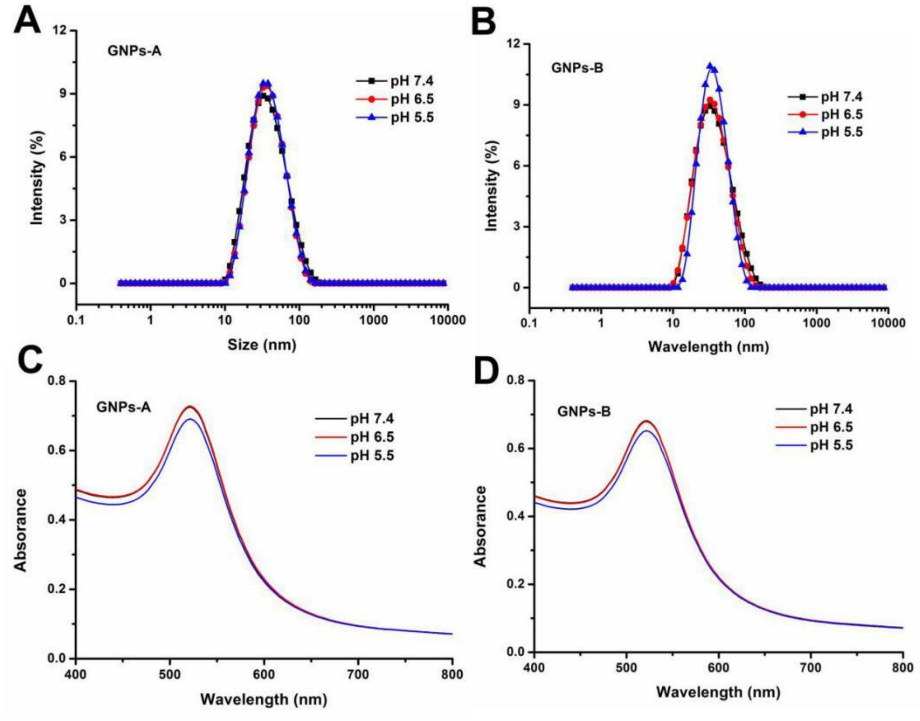

Figure S8. The size and UV-vis spectra of GNPs-A and GNPs-B at 7.4, 6.5 and 5.5 pH value.

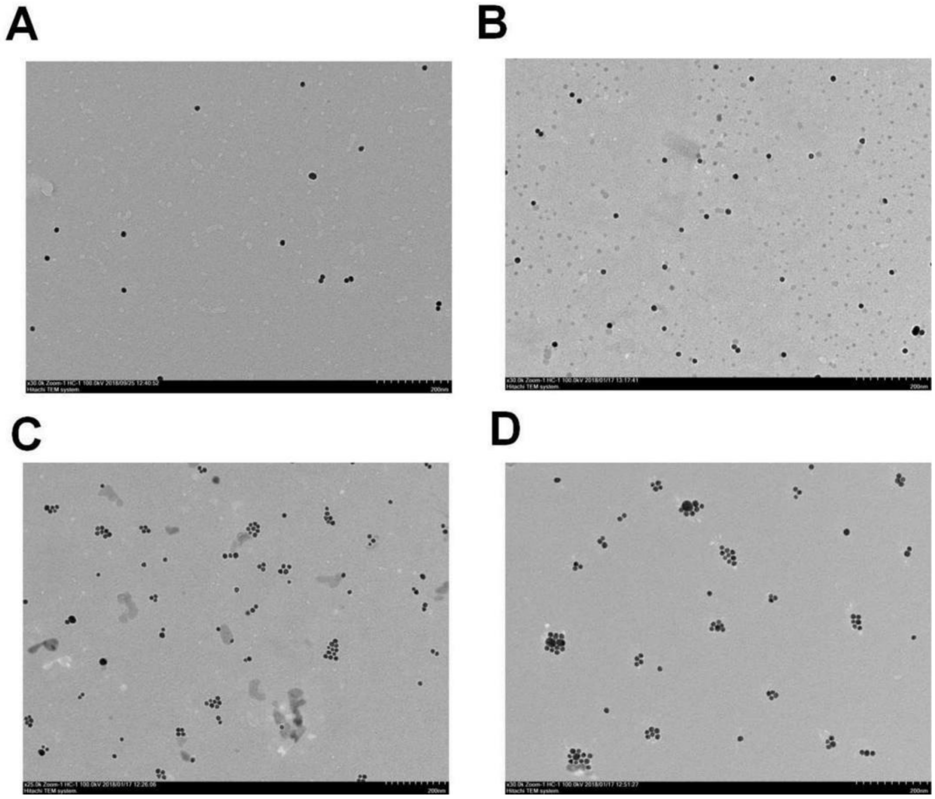

Figure S9. The TEM images of GNPs-A and GNPs-B at 7.4 (A and C) and 5.5 pH value (B and D).

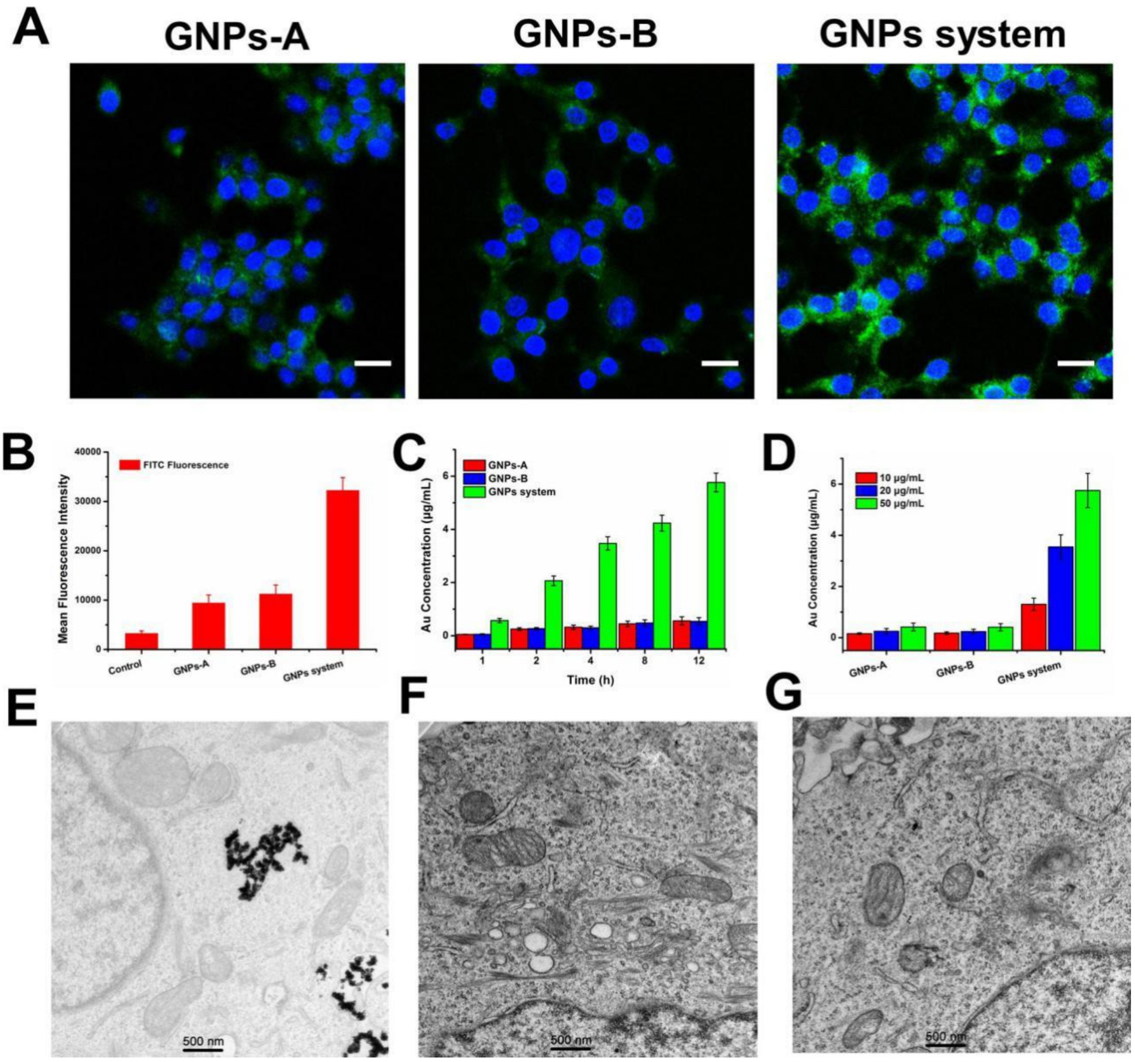

Figure S10. In vitro evaluation of cellular uptake. Fluorescence image of MCF-7 cells incubated with GNPs system-Fitc and other control groups for 4 h (A). Quantitative analysis of cell uptake by MCF-7 cells treated with various of GNPs after treatment for 4 h by using flow cytometry (B). Quantitative analysis of cell uptake by MCF-7 cells treated with various of GNPs after treatment for different incubated time by using ICP-AES (C), and the cell uptake behavior of GNPs at different GNPs concentrations after incubation of 12 h detected by ICP-AES (D). TEM images of MCF-7 cells incubated with GNPs system (E), GNPs-A (F), and GNPs-B (G) for 12 h.

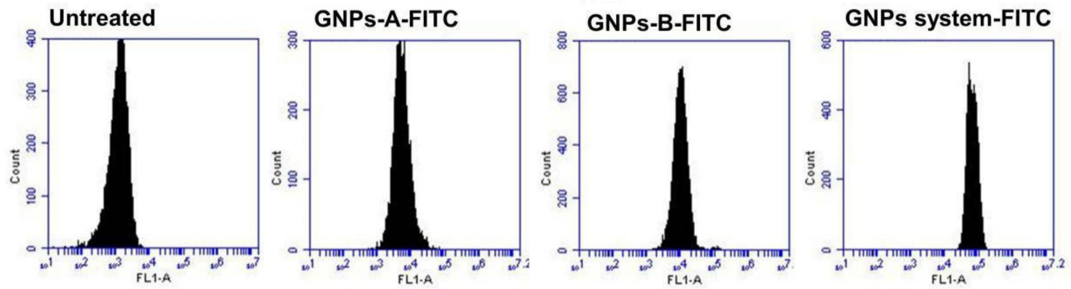

Figure S11. The Quantitative analysis of cell uptake by MCF-7 cells treated with various GNPs formulations after treatment for 4 h followed by flow cytometry.

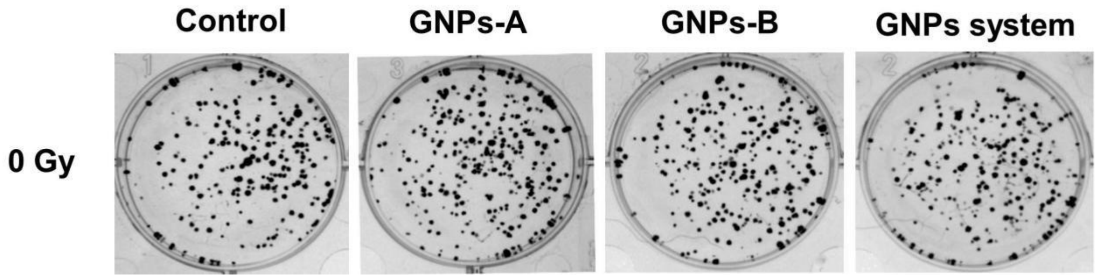

Figure S12. Representative photographs of colony formation of various groups without irradiation.

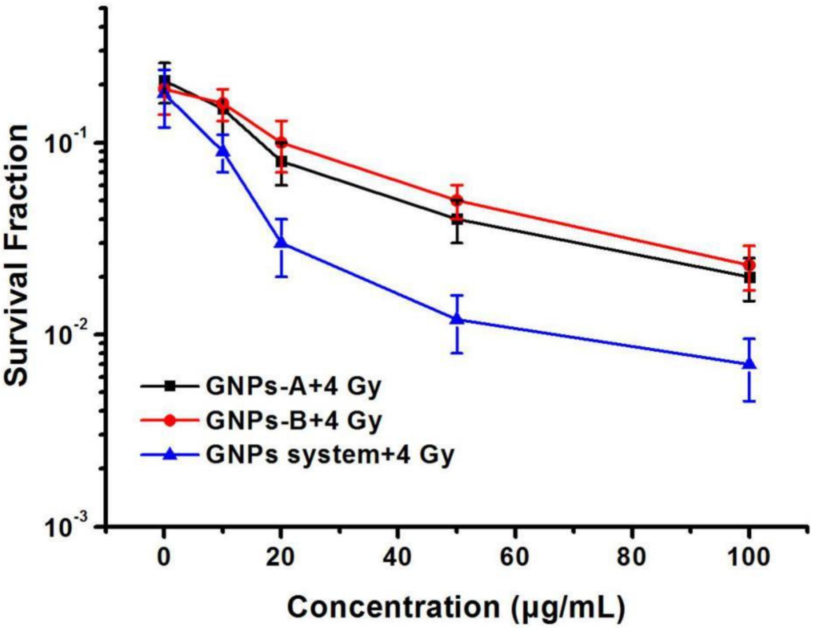

Figure S13. The colony formation curve of various of GNPs groups of various concentrations under 4 Gy radiation.

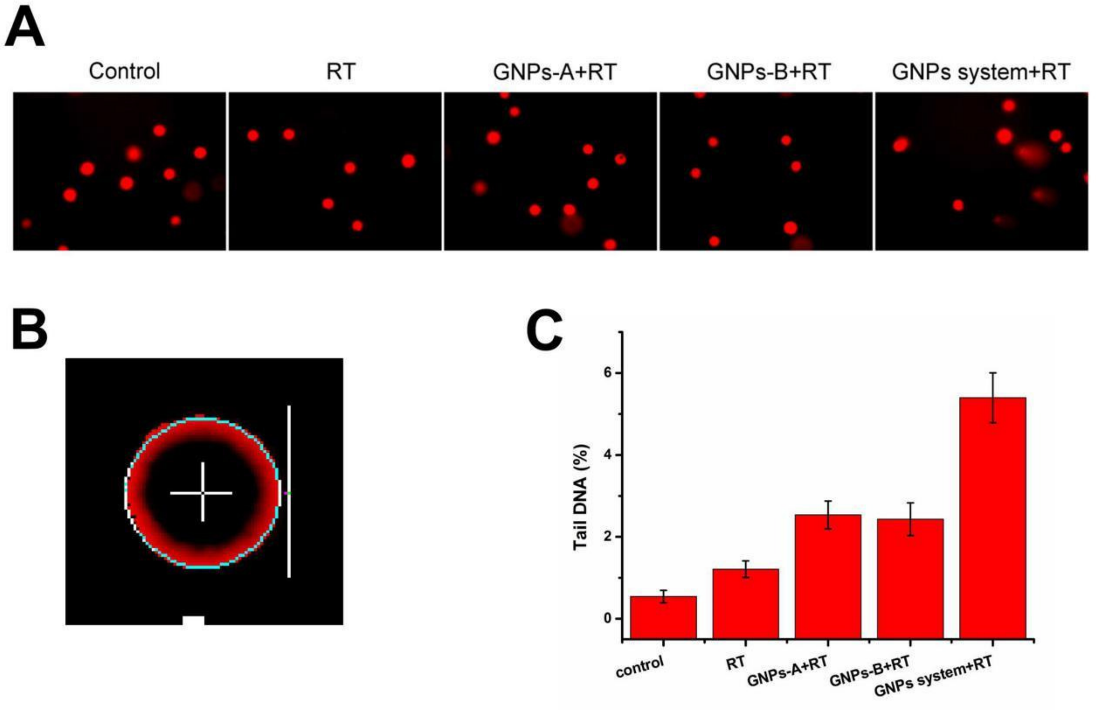

Figure S14. Imaging of DNA fragmentation by using comet assay in MCF-7 cells with different GNPs treatments under 4 Gy irradiation (A). The result of comet assay with CASP analysis in MCF-7 cells without any treatment (B). Tail DNA ratio of various of treatment under 4 Gy irradiation (C).

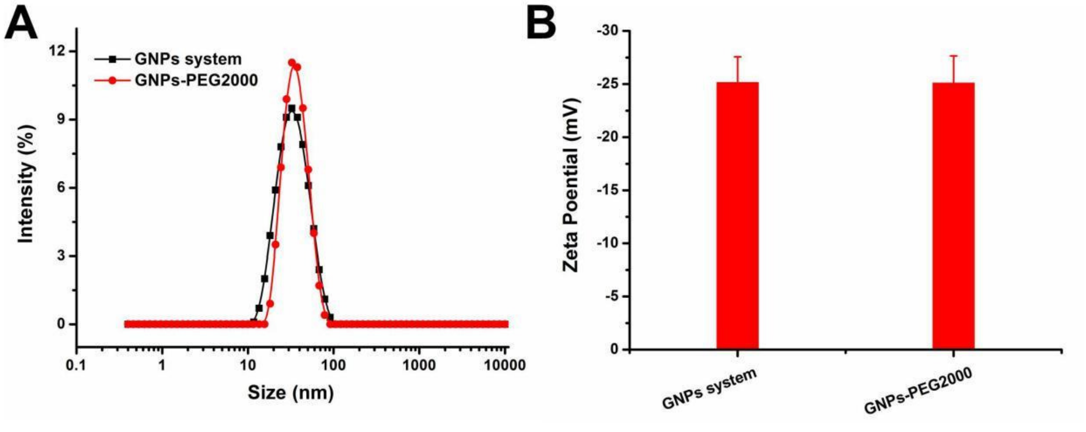

Figure S15. The size distribution and zeta potential of GNPs system and GNPs-PEG2000.

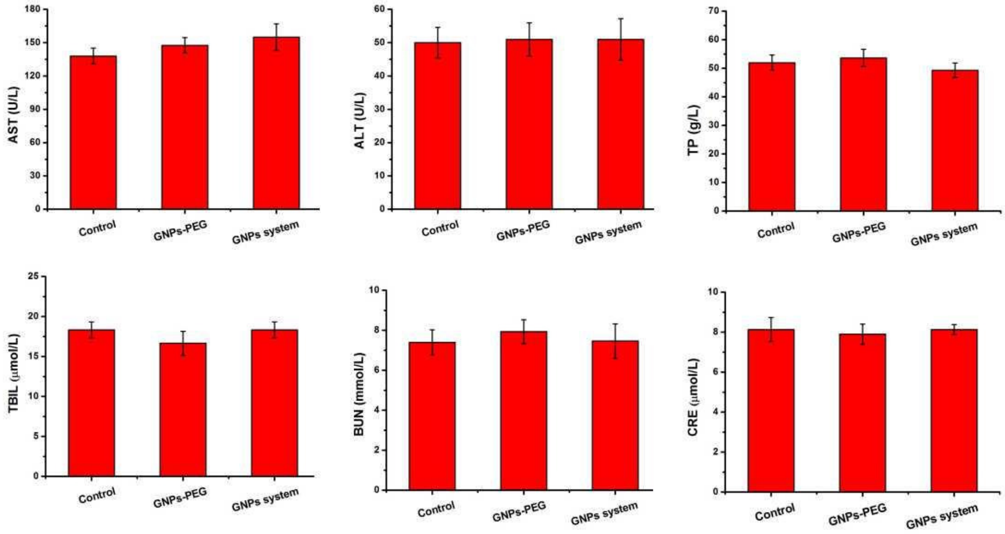

Figure S16. Hematology results of BALB/c mice after 24 h i.v. injection of GNPs system and GNPs-PEG2000 of 60 mg/kg/dose, respectively.

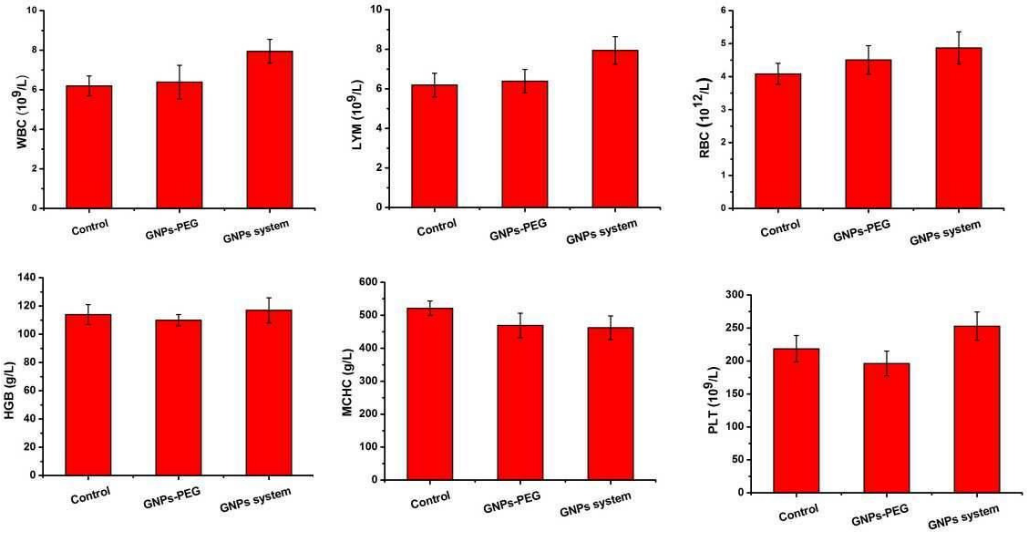

Figure S17. Blood biochemistry data of BALB/c mice after 24 h i.v. injection of GNPs system and GNPs-PEG2000 of 60 mg/kg/dose, respectively.

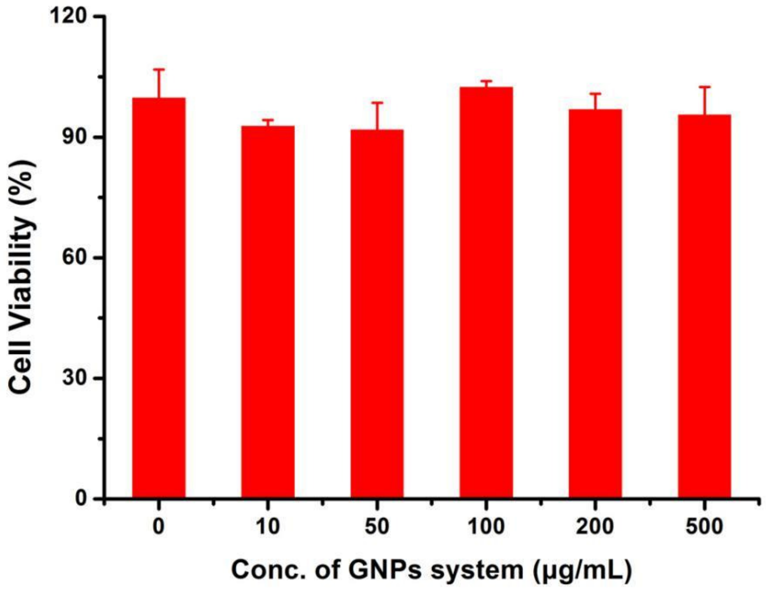

Figure S18. Relative viabilities of MCF-7 cells incubated with different concentrations of GNPs system for 24 h.

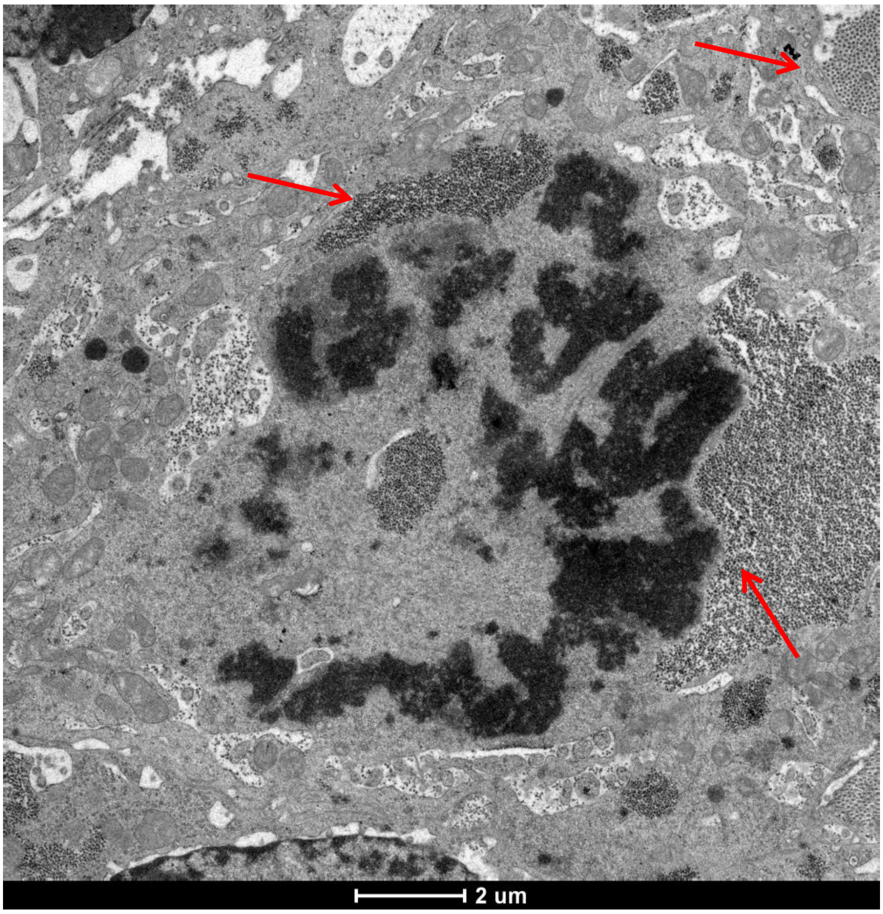

Figure S19. GNPs aggregates formed in the tumor tissues after 24 injection of GNPs system at the lower magnification.

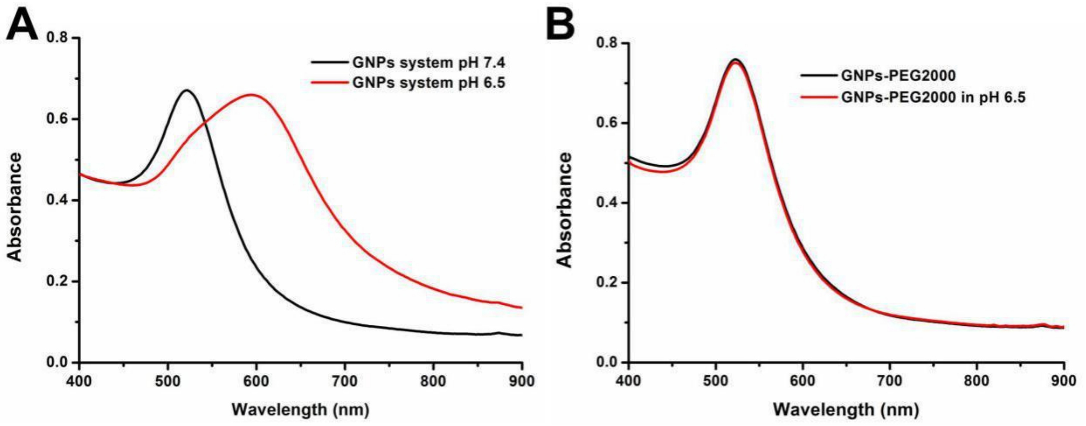

Figure S20. UV-Vis absorption spectra of GNPs system and GNPs-PEG2000 in pH 7.4 and pH 6.5 value.

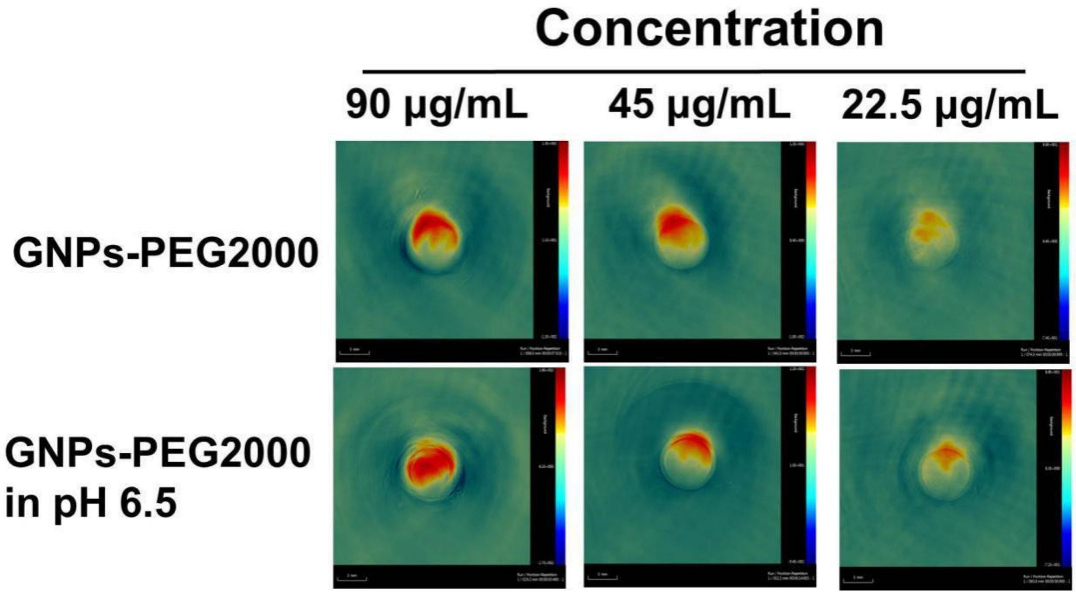

Figure S21. the in vitro PA imaging of GNPs-PEG2000 at concentrations of 90, 45, 22.5 μg/mL in pH 7.4 and 6.5 values.

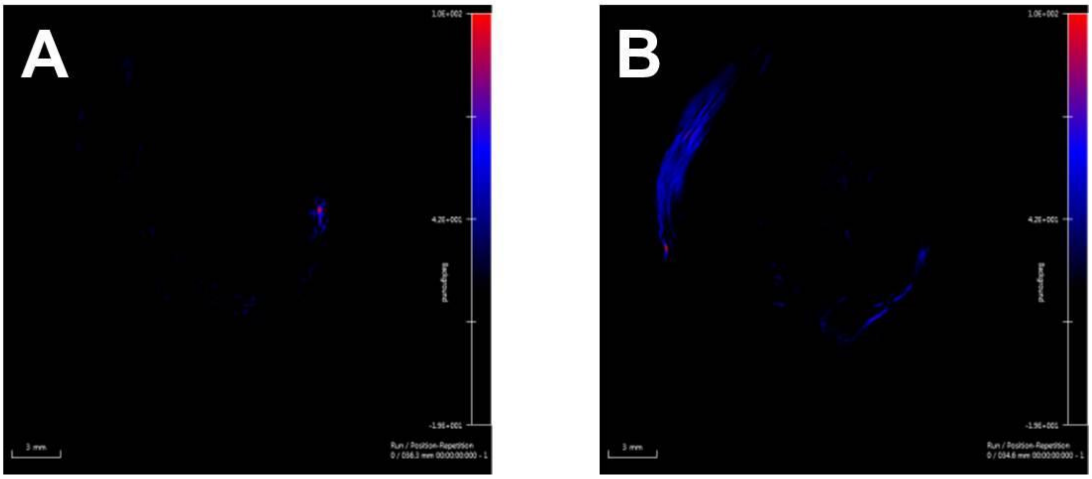

Figure S22. The in vivo PA imaging of tumor bearing mice before i.v. GNPs system and GNPs-PEG2000 of injection.

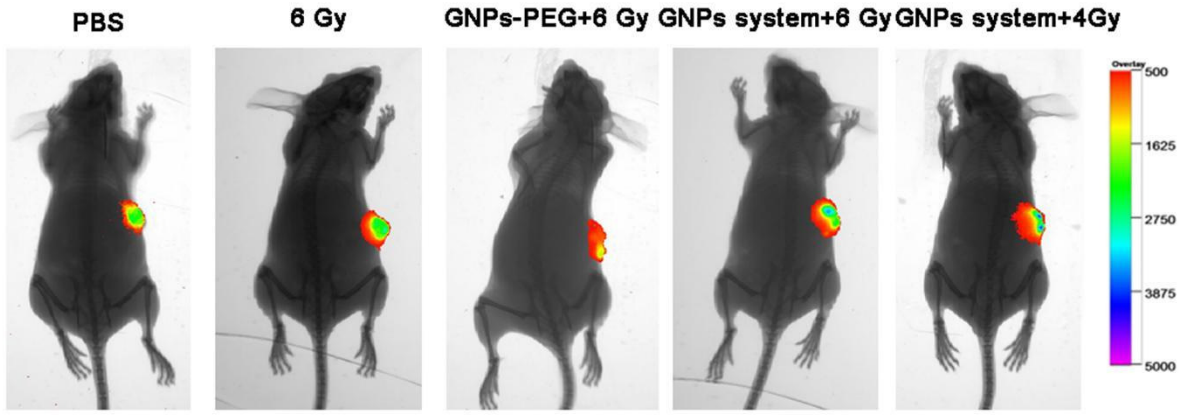

Figure S23. Bioluminescent imaging on MCF-7-luciferase tumor-bearing mice 20 days before the treatments.

Table S1. Statistical data of the cell cycle distribution ratios of MCF-7 cells after various treatments (various GNPs formulations and/or 4 Gy irradiation)

Sample	G1 (%)	S (%)	G2/M (%)
Control	45.5±1.3	23.0±0.1	31.5±1.4
GNPs-A	44.8±0.6	24.0±2.5	31.2±1.9
GNPs-B	44.6±2.4	22.2±0.8	33.3±1.5
GNPs system	43.1±0.8	18.9±0.4	37.9±1.1

RT	62.6±0.5	12.6±1.9	24.8±3.6
RT+GNPs-A	62.9±2.0	14.8±0.8	22.3±2.3
RT+GNPs-B	63.4±0.7	16.8±1.6	19.8±0.9
RT+GNPs system	64.9±0.4	18.9±1.8	16.3±1.3

2. Experimental Section

Synthesis of GNPs system. The gold nanoparticles (GNPs) were firstly synthesized by using the citrate-reduction method, and its diameter and morphology was characterized by dynamic light scattering (DLS) and transmission electron microscope (TEM). Secondly, these two peptides (Asp-Asp-Asp-Asp-Asp-Cys (peptide A) and Lys-Gly-Gly-Lys-Gly-Gly-Lys-Cys (peptide B)) were synthesized by using solid phase synthesis and purified by reversed phase high-performance liquid chromatography (RP-HPLC), and their purity and identity were confirmed by LC-MS. And then, 100 mg of peptide B and 206 mg of 2,3-Dimethylmaleic anhydride (DA) were co-dissolved in PBS (pH 8.0) and slowly stirred for 24 h at room temperature. The excessive DA was removed by dialysis method. The white powder of DA grafted peptide B was then obtained by ultra-low temperature freeze-drying technology, and its structure was characterized by using ^{1}H NMR (Varian INOVA). Lastly, the peptide A (1.75 mg) and DA grafted peptide B (2.5 mg) was conjugated onto GNPs (180 μg/mL, 10 mL) at pH 8.0 for stirring 24 h at room temperature, respectively. The GNPs-A and GNPs-B were purified by centrifugation at 6000 rpm (10 min) for three times. Subsequently, the GNPs system was finally obtained by mixing GNPs-A and GNPs-B with the same concentration of GNPs.

Characterization of GNPs system. In order to verify the strategy of acid triggered aggregation by the means of charge reverse of DA grafted peptide B, the DA grafted peptide B was dissolved in D_2O/DCL at pH 6.5 for 5 min, and its structure was characterized by ^{1}H NMR (Varian INOVA). After the GNPs system being obtained, its ultraviolet visible (UV-vis) absorption spectrum, DLS and TEM of GNPs system were firstly characterized to verify the preparation of GNPs system by using VARIOSKAN FLASH microplate reader (THERMO SCIENTIFIC), UV-vis (Purkinje General), DLS (Malvern Zetasizer Nano ZS) and TEM (JEM-2100F). Meanwhile, the in vitro stability of GNPs system was investigated by using the UV-vis and DLS at predetermined time point of 0, 0.5, 1, 2, 6, 12, 24 h. Afterwards, to determine the pH induced aggregation of GNPs system, the UV-vis spectrum, size and morphology of GNPs system at pH 6.5 was measured by UV-vis, DLS and TEM. To further confirm the charge reverse of DA grafted peptide, the zeta potential of GNPs, GNPs-A, GNPs-B and GNPs system at pH 7.4 and pH 6.5 were monitored by DLS (Malvern Zetasizer Nano ZS), respectively. Meanwhile, in order to investigate the stability of GNPs agggregates in neutral pH, the UV-vis spectrum of GNPs aggregates formed in pH 6.5 was also recorded after adjusting back to pH 7.4 value. At last, in order to illustrate the specificity of GNPs system, the size and UV-vis spectrum of single GNPs-A and GNPs-B were detected at pH 7.4, pH 6.5, pH 5.5, respectively.

In Vitro Evaluation of Cellular Uptake.

Cell culture. MCF-7 cells were cultured by Dulbecco's modified Eagle's medium (DMEM) plus 10 % fetal bovine serum (FBS), streptomycin (0.1 mg/mL), and penicillin (100 U/mL) at 37 °C in a humidified incubator with 5% CO_2.

Confocal image. Firstly, we used a fluorescence labeled GNPs system to investigate the cellular uptake behavior via confocal microscopy. 0.05 mg of FITC-PEG_{1000}-SH was modified with GNPs system (10 mL) according to the preparation of GNPs system. Secondly, MCF-7 cells were seeded in the confocal culture dish with a density of 10^5 cells in each dish and cultured for 24 h at 37 °C in a humidified incubator with 5% CO_2. Then the cell culture medium was replaced by GNPs system (50 μg/mL) and incubated with cells for 4 h, and the individual GNPs-A and GNPs-B as the control. After 4 h incubation, the cells were fixed by 4% paraformaldehyde for 30 min. After rinsing with PBS, the cells were stained with DAPI for 15min. The cells were infiltrated in the PBS solution and imaged by confocal microscope (Zeiss, LSM710).

Flow cytometer analyses. Cells were seeded into 6-well plates with a density of 2×10^5/well and incubated for 24 h at 37 °C in a humidified incubator with 5% CO_2. Cells were further incubated with GNPs-A, GNPs-B, GNPs-system (50 μg/ml) for 4 h, which PBS was used as the blank control. After washing three times with cold PBS, cells were collected for quantitative detected by flow cytometer on a FACS Calibur (BD, Accuri C6).

Quantitative determination by ICP-AES. MCF-7 cells were seeded in 6-well plates about 2×10^5 per well and incubated for 24 h. Then cell culture medium was replaced by various of GNPs formulations (single GNPs-A and GNPs-B, GNPs-system) in DMEM (10 μg/mL, 20 μg/mL, 50 μg/mL) to the plate and incubated for 12 h. Meanwhile, cells were incubated with GNPs-A, GNPs-B, GNPs-system (50 μg/mL) for a series of predetermined time (1 h, 4 h, 8 h, 12 h). After that, cells were washed by cold phosphate buffered saline (PBS) solution and then collected within EP tube. Then the cells were lysed and treated with aqua regia (HCl/HNO3 =

1:3, volume ratio) for 2 h, and then the Au concentration of each wells were measured by using ICP-AES (ThermoFisher iCAP7400).

Internalization of GNPs Detected by TEM Analysis. MCF-7 cells were seeded in 6-well plates about 2×10^5 per well and incubated for 24 h. Cell culture medium was replaced by various of GNPs formulations (GNPs-A, GNPs-B, GNPs-system) in DMEM (50 μg/mL) to the plate and incubated for 12 h. Cells were washed with PBS and trypsizined, centrifuged, and then fixed with 2.5% glutaraldehyde. After 2 h fixation at 4 °C, the samples were washed with PBS (0.02 M, pH 7.4) three times. Then the samples were fixed with 1% perosmic oxide for 2 h at 4 °C. After being washed by water, the samples were dehydrated in an alcohol series, embedded, and sliced with a thickness of 50 to 70 nm. The morphology of GNPs system within MCF-7 cells was imaged by using a TEM (Hitachi H-600, Japan) at 200 kV.

In Vitro Sensitization Efficiency Study.

Colony formation assay. MCF-7 cells were cultured in 6-well plates at a density of 10^3 per well for 24 h, the cell culture medium was replaced by GNPs-A, GNPs-B, GNPs-system (20 μg/mL) and incubated for another 24 h. After washing the excessive GNPs by cold PBS, the cells were irradiated at 0 Gy, 2 Gy, 4 Gy, 6 Gy by using a gamma radiation of ^{137}Cs (photon energy 662 keV) with an activity of 3600 Ci. The cells were incubated for 7 days and then stained with 0.25 % crystal violet. The colonies with more than 50 cells were counted to calculate the surviving fraction, and the radio-enhancing effect was assessed by calculating Sensitizer Enhancement Ratio (SER) according to previous report,[1] that is, a 10 % survival fraction was set to obtain the required dose of different agents. To further verify the dose dependant radiosensitive efficiency of GNPs system, different concentration (0 μg/mL, 10

μg/mL, 20 μg/mL, 50 μg/mL, 100 μg/mL) of GNPs system was cultured with cells, respectively, and the individual GNPs-A and GNPs-B were used as the control groups. Whereafter, we calculated the SER10 value of GNPs system at the concentration of 20 μg/mL, 50 μg/mL, 100 μg/mL by using the colony formation assay under a 2 Gy, 4 Gy, 6 Gy irradiation with a gamma ray.

DNA breakage detection. For γ-H2AX immunofluorescence analysis, MCF-7 cells were seeded in confocal cell culture dish and incubated with various of GNPs in DMEM (50 μg/mL) for 12 h. After excessive GNPs were washed by cold PBS, the cells were irradiated with gamma rays under 4 Gy. After additional culturing for 1 h, the cells were fixed by 4% paraformaldehyde for 30 min. The protocol of immunofluorescence analysis referenced the published studies.[2] After being stained with DAPI for 15 min, cells were observed and imaged by using fluorescence microscope. Secondly, for comet assay detection, MCF-7 cells were seeded in 6-well plates with a density of 2×10^5 and incubated with GNPs at a concentration of 50 μg/mL for 24 h. The cells were irradiated with gamma rays at dose of 4 Gy after being removed the excessive GNPs. Then the cells were collected, and the DNA breakage was detected by using a single cell gel electrophoresis. The microscope slides were covered with agarose, after the solidification, 30 μl of cells (10^5 cells) and 70 μl of low-melting-point agarose were mixed and added to each slide. The slides were placed in the lysed buffer for 2.5 h, placed in the electrophoretic liquids for 20 min and electrophoresis at 30 V for 20 min, after being neutralized for 20 min and washed with PBS, the comets on the slides were stained with ethidium bromide. The DNA damage was analyzed by using comet assay software project (CASP).

Cell cycle experiment. The variation of cell cycle which treated with GNPs system was monitored by cell cycle detection kit. MCF-7 cells were seeded in 6-well plates about 10^5 per well and incubated for 24 h. Then cells were treated with GNPs system in DMEM (50 μg/mL) for 24 h. After excessive nanoparticles removed by cold PBS washing, the cells were irradiated at 0 Gy or 4 Gy. After 24 h incubation, the cells were collected, washed with PBS and fixed with pre-cooled 70% ethanol solution and stored at 4°C. Prior to staining, the ethanol solution was removed and the cells were washed with PBS, cells were incubated with 100 μL of RNase A at 37 °C for 30 min. Then 500 μL of 50 μg/mL Propidium iodide (PI) was added and the mixture was incubated at 37 °C for 10 min. Finally, the cell cycle was analyzed with a flow cytometer in FL-2 channel (Ex/Em = 488 nm/630 nm).

Cell Apoptosis Analysis. We compared the apoptosis-inducing capabilities of single GNPs and GNPs system after 4 Gy irradiation by using the Annexin V–FITC/PI apoptosis detection kit. Firstly, MCF-7 cells (10^6 cells) of control group, RT only group, GNPs-A+RT group, GNPs-B+RT group and GNPs system+RT group were trypsinized and re-suspended in 500 μL of binding buffer. Secondly, 5 μL of Annexin V–FITC (20 μg/mL) and 5 μL of PI (50 μg/mL) were contiguously added into the above buffers and then incubated for another 15 min at room temperature in dark. Finally, cell apoptosis in each group were analyzed by flow cytometry .

In vivo evaluation of GNPs system. Given the polyethylene glycol (PEG2000) is approved by FDA and could be used as a golden standard in pharmaceutics,[3] so the PEG2000 conjugated GNPs (GNPs-PEG2000) was used as the control. BALB/c mice (20 g) were divided into two groups of GNPs system and GNPs-PEG2000, and each group had eight

animals. Mice were intravenously injected with GNPs system and GNPs-PEG2000 at a dose of 60 mg/kg, respectively. Blood samples were collected at a different time points (0.25, 0.5, 1, 2, 4, 8, 12, 24 h). And after 24 h injection, the main tissues including liver, kidney, spleen, heart, and lung were dissected, the Au concentration in blood and different tissues were quantitative measured by ICP-AES. Meanwhile, the in vivo toxicity of GNPs system after being injected 24 h was analyzed by using the hematology, blood biochemistry analysis and histology. Briefly, the whole blood samples were collected from eyes after 24 h injection, 70 μL of blood sample was collected into heparinized tube for hematological analysis with automatic blood analyzer (Celltace, Japan). 400 μL of blood samples were centrifuged at 4000 rpm for 5 min and serum was harvested for biochemical analysis via automatic biochemical analyzer (Vitalab, Holland). Lastly, the liver, spleen and kidney was harvest and fixed in 4 % neutral buffered formalin for 24 h, and the Haematoxylin & Eosin (H&E) assays were performed according to the standard protocols.[4]

Tumor retention of GNPs system. MCF-7 cells (10^6) in 0.2 mL of PBS were injected subcutaneously into the right rear flank area of BALB/c nude mice (20 g). When the tumor size reached to ~100 mm^3, GNPs system and GNPs-PEG2000 were injected via the tail vein at a GNPs dose of 60 mg/kg. Tumors were collected for measuring their Au concentration by ICP-AES and observing the GNPs retention within the tumor by using TEM analysis at 24, 48 and 72 h post injection. For ICP-AES measurement, the main tissues and tumors were washed with PBS buffer and lyophilized for 24 h, and blood can be lyophilized directly. The dried samples were mashed and dissolved in aqua regia (2 mL for liver and 1 mL for all others) for 24 h. Tissue debris was removed by centrifugation at 12 000 rpm for 10 min. The Au content

of biological samples was quantitative detected by ICP-AES. For TEM observation of GNPs within tumors, Tumors were fixed in 2.5 % glutaraldehyde (in 0.1 M phosphate buffer, pH 7.0), and then fixed with 1% perosmic oxide for 2 h at 4 °C. After being washed by water, the samples were dehydrated in an alcohol series, embedded, and sliced with a thickness of 50 to 70 nm, and lastly observed by TEM.[5]

Photoacoustic Imaging of GNPs system. Various concentration of GNPs system (90, 45, 22.5 μg/mL) at pH 7.4 and pH 6.5 were embedded in agar gel cylinders to create in vitro PA imaging phantoms which were studied in a multispectral optical tomography system (MSOT inVision 128, iThera medical, Germany), and the same concentrations of GNPs-PEG2000 at pH 7.4 and pH 6.5 were used as a control. For in vivo PA imaging, tumor bearing nude mice were intravenous injected with GNPs system and GNPs-PEG2000 (30 mg/kg, n=3). At the predetermined time (0, 1, 3, 6, 12, 24, 48 h), the mice were first anaesthetized with 3% isoflurane, and ultrasound gel was painted on mice skin of the tumor field. The PA imaging of multiwavelength (690, 715, 730, 760, 800, 815, and 850 nm) was captured under the same condition with a step size of about 0.3 mm step distance along the long axis of tumors, and the maximum contrast PA signal was obtained when the excitation wavelength was 680 nm, and the oxygenated and deoxygenated hemoglobin signals at excitation wavelengths of 850 nm and 750 nm were deducted, respectively.[6]

In Vivo Radio-sensitization Evaluation of GNPs system. Luciferase labeled MCF-7 cells were used to build breast tumor bearing mice. Cells were injected subcutaneously into the right rear flank area of BALB/c nude mice (20 g). When the tumor size reached to ~100 mm^3, the mice were divided into five groups (n=6), including the untreated group (PBS), PBS with

6 Gy radiated group, GNPs-PEG2000 injected with 6 Gy radiation group, and GNPs system injected with 4 Gy radiation group and with 6 Gy radiation group. The GNPs system and GNPs-PEG2000 were injected via tail vein with a GNPs dose of 60 mg/kg. After injection of 24 h, mice were irradiated with gamma rays for the designed radiation dose. The tumor volume and body weight were measured every other day during the treatment. On day 20, the MCF-7-luciferase tumor bearing mice of each groups were received i.p. injection of 150 mg of D-luciferin/kg body weight (D-luciferin, firefly, potassium salt, SynChem, Inc.). After ten minutes for anesthetized with 4 % chloral hydratelater, bioluminescent imaging of MCF-7-luciferase tumor was imaged by using KODAK IS in vivo FX system.[7] After sacrificed all the mice, the tumor weight (g) of each groups was recorded. Ultimately, excised tumors of each group were used to pathological analysis, TUNEL assay through the HE staining, tunel staining, which was according to the protocol of the corresponding detection kit and protocol, respectively.[7]

References

[1] C. Y. Y. Yu, H. Xu, S. Ji, R. T. K. Kwok, J. W. Y. Lam, X. Li, S. Krishnan, D. Ding, B. Z. Tang, Adv. Mater. **2017**, 29, 1606167.

[2] M. Li, Q. Zhao, X. Yi, X. Zhong, G. Song, Z. Chai, Z. Liu, K. Yang, ACS Appl. Mater. Interfaces **2016**, 8, 9557.

[3] a) H. Cabral, Y. Matsumoto, K. Mizuno, Q. Chen, M. Murakami, M. Kimura, Y. Terada, M. R. Kano, K. Miyazono, M. Uesaka, N. Nishiyama, K. Kataoka, Nat. Nanotechnol. **2011**, 6,

815; b) J. S. Suk, Q. Xu, N. Kim, J. Hanes, L. M. Ensign, Adv. Drug Delivery Rev. **2016**, 99, 28.

[4] a) C. Yang, L. Chu, Y. Zhang, Y. Shi, J. Liu, Q. Liu, S. Fan, Z. Yang, D. Ding, D. Kong, J. Liu, ACS Appl. Mater. Interfaces **2015**, 7, 2735; b) Y. Zhang, F. Huang, C. Ren, L. Yang, J. Liu, Z. Cheng, L. Chu, J. Liu, ACS Appl. Mater. Interfaces **2017**, 9, 13016.

[5] a) S. M. Aditi Mulgaonkar, William Silvers, Gedaa Hassan, Xiankai Sun, Yaowu Hao, and Weihua Mao., J. Biomed. Nanotechnol. **2017**, 133, 566; b) X. Liu, Y. Chen, H. Li, N. Huang, Q. Jin, K. Ren, J. Ji, ACS Nano **2013**, 7, 6244; c) V. C. María A. Escudero-Francos, Pedro González-Menéndez, Rosana Badía-Laíño, Marta E. Díaz-García, Rosa M. Sainz, Juan C. Mayo, and David Hevia, J. Biomed. Nanotechnol. **2017**, 13, 167; d) S. Ruan, C. Hu, X. Tang, X. Cun, W. Xiao, K. Shi, Q. He, H. Gao, ACS Nano **2016**, 10, 10086.

[6] a) J. Han, J. Zhang, M. Yang, D. Cui, J. M. de la Fuente, Nanoscale **2016**, 8, 492; b) G. Lv, W. Guo, W. Zhang, T. Zhang, S. Li, S. Chen, A. S. Eltahan, D. Wang, Y. Wang, J. Zhang, P. C. Wang, J. Chang, X.-J. Liang, ACS Nano **2016**, 10, 9637; c) L. Tan, J. Wan, W. Guo, C. Ou, T. Liu, C. Fu, Q. Zhang, X. Ren, X.-J. Liang, J. Ren, L. Li, X. Meng, Biomaterials **2018**, 159, 108; d) S. Zhang, W. Guo, J. Wei, C. Li, X.-J. Liang, M. Yin, ACS Nano **2017**, 11, 3797.

[7] J. Liu, J. Liu, L. Chu, Y. Zhang, H. Xu, D. Kong, Z. Yang, C. Yang, D. Ding, ACS ACS Appl. Mater. Interfaces **2014**, 6, 5558.
